# Real world treatment sequences and outcomes for metastatic renal cell carcinoma

**DOI:** 10.1371/journal.pone.0294039

**Published:** 2023-11-22

**Authors:** Gu-Shun Lai, Jian-Ri Li, Shian-Shiang Wang, Chuan-Shu Chen, Chun-Kuang Yang, Chia-Yen Lin, Sheng-Chun Hung, Kun-Yuan Chiu, Shun-Fa Yang

**Affiliations:** 1 Institute of Medicine, Chung Shan Medical University, Taichung, Taiwan; 2 Division of Urology, Department of Surgery, Taichung Veterans General Hospital, Taichung, Taiwan; 3 Department of Medicine and Nursing, Hungkuang University, Taichung, Taiwan; 4 Department of Applied Chemistry, National Chi Nan University, Nantou, Taiwan; 5 Department of Medical Research, Chung Shan Medical University Hospital, Taichung, Taiwan; IRCCS Giovanni Paolo II Cancer Hospital, ITALY

## Abstract

**Objectives:**

The treatment landscape for metastatic renal cell carcinoma changed a lot in the last few years. This study aimed to assess the treatment sequences and outcomes for metastatic renal cell carcinoma in a real-world setting.

**Materials and methods:**

We enrolled patients with metastatic renal cell carcinomawho received first-line systemic treatment with tyrosin kinase inhibitors monotherapy, ipilimumab plus nivolumab, or pembrolizumab plus axitinibbetween January2009 and May 2023 on the database of TriNetX network. Overall survival, time on treatment and time to next treatment were evaluated using Kaplan-Meiermethod.

**Results:**

Totally, 4183 received tyrosine kinase inhibitor monotherapy, 1555 received ipilimumab plus nivolumab, and 559 received axitinib plus pembrolizumab. Median time on treatment was 2.5 months for the tyrosine kinase inhibitor monotherapy cohort, 5.4 months for the ipilimumab plus nivolumab cohort, and 8.3 months for the pembrolizumab plus axitinib cohort. Median time to next treatment was 16.6 months for both the tyrosine kinase inhibitor monotherapy and ipilimumab plus nivolumab cohorts, and 22.1 months for the pembrolizumab plus axitinib cohort. Median overall survival was 42.2 months for the tyrosine kinase inhibitor monotherapy cohort, 39.7monthsfor the ipilimumab plus nivolumab cohort, and not reached for the pembrolizumab plus axitinib cohort. In comparison with the tyrosine kinase inhibitor monotherapy cohort, patients in the pembrolizumab plus axitinib cohort showed survival benefit (log-rank *p* = 0.0168) in overall survival, but not the case in the ipilimumab plus nivolumab cohort.

**Conclusion:**

There was a trend toward using first-line immuno-oncology based therapy for patients with metastatic renal cell carcinoma in a real-world practice. Axitinib plus pembrolizumuab cohort had survival benefits over tyrosine kinase inhibitor and ipilimumab plus nivolumab cohorts, while patients in the ipilimumab plus nivolumab cohort had more distant metastases and comorbidities.

## Introduction

With the introduction of tyrosine kinase inhibitor (TKI) and immune-oncology (IO) agent, the first-line systemic therapy for advanced renal cell carcinoma (RCC) has evolved in recent years [1–9]. The current European Association of Urology Guidelines recommends the combined treatment with IO-TKI, IO-IO, and TKImonotherapy as the first-line management for advanced RCC [10].

Several phase III clinical trials demonstrated the efficacy of such combination therapies, including CheckMate 214 (Ipilimumab + Nivolumab versus Sunitinib), KEYNOTE-426 (axitinib + pembrolizumab versus sunitinib),CHECKMATE-9ER (cabozantinib + nivolumab versus sunitinib), CLEAR (pembrolizumab + lenvatinib versus sunitinib), and JAVELINtrial (avelumab + axitinib versus sunitinib) [2–7]. However, only a few studies reported real-world data regarding treatment pattern and clinical outcome of combination therapies on RCC [11–14]. In this era of IO and TKI targeted therapy, it is important to understand the treatment efficacy and sequence of these agents. Here we reported treatment trends, sequences and clinical outcomes for patients with metastatic RCC (mRCC) receiving first-line IO or TKI based agents in a real-world setting.

## Materials and methods

### Data source

We conducted a retrospective analysis of patients with mRCC between January 1, 2009 and May 30, 2023on the database of TriNetX network, a global health research network that provides real-world clinical data of ≥250 million patients in 120 healthcare organizations. In the present study, we specifically used the US Collaborative Network, which includes 57 healthcare organizations in the US.

### Study design and population

We enrolled patients with mRCC aged ≥18 years old receiving first-line systemic therapies between January 1, 2009 and May 30, 2023. To confirm the diagnosis of distant metastasis, these patients were identified using the International Classification of Diseases, tenth edition, Clinical Modification (ICD-10-CM): ICD-10-CM C64, as well as ICD-10-CM: C78.0, C78.7,C79.3, or C79.5 to confirm the diagnosis of distant metastasis. The index date was set at the date of initiation of first-line systemic treatment for mRCC. Included patients must have ≥2clinical visits and a follow-up duration of≥6 months after the index date.

In the present study, the first-line systemic treatments included TKI monotherapy (sunitinib, pazopanib), ipilimumab plus nivolumab, and axitinib plus pembrolizumab. A combination treatment was defined as any treatments given within one month after the index date.

### Outcome measurement

The clinical outcomes we analyzed were the following 3 items: time on treatment (ToT), time to next treatment (TNT), and overall survival (OS). ToT was defined as the duration between the index date and the date when a new treatment was introduced, on the death of the patient, patient’s medical record ended, the date of the last administration of first-line treatment if there was a gap of ≥4 months when a patient did not receive any treatment, or censored at the date of the last administration of first-line therapy, whichever had taken place first. TNT was calculated from the index date to the date of initiation of second-line treatment, or censored at the date of the last administration of first-line therapy, whichever had taken place first. OS was defined as duration between the index date and the date of death from any cause or censored at the date of the study end, whichever had taken place first.

### Statistical analyses

Patient baseline characteristics for continuous variables were expressed as mean and standard deviation(SD) and for categorical variables as number and percentage. Inter-group differences were evaluated using Student’s t test for continuous variables, and chi-square test for categorical variables. Survival analysis was calculated using Kaplan-Meier method with a log-rank test to assess the inter-group differences on ToT, TNT and OS. Statistical analyses were conducted on the TriNetX platform and statistical significance was set at *p*<0.05.

### Ethics in research

This study was approved by the institutional review board (IRB) of Taichung Veterans General Hospital (IRB number: SE:22220A). The operation was performed in accordance with national regulations and the Helsinki Declaration.

## Results

### Baseline characteristics

We identified a total of 6297 patients with mRCC. Of them, 4183 received TKI monotherapy, 1555 received ipilimumab plus nivolumab, and 559 received axitinib plus pembrolizumab. Within the TKI monotherapy cohort, 2110 (50.4%) received sunitinib and 2073 (49.6%) received pazopanib.

Patient characteristics are showed in Table 1. Compared with TKI monotherapy cohort, patients in the ipilimumab plus nivolumab cohort experienced significantly more instances of distant metastases, including lung (*p*< 0.0001), liver (*p* = 0.0002), bone (*p*< 0.0001), and brain metastasis (*p* = 0.0064). Additionally, more patients in the ipilimumab plus nivolumab cohort had comorbidity compared with TKI montheray cohort, including diabetes mellitus, hypertension, cerebrovascular disease, ischemic heart disease (all with *p*< 0.0001). Patients in the axitinib plus pembrolizumab cohort were older (*p* = 0.01) and had few white people (*p* = 0.0213) in comparison with the TKI monotherapy cohort.

**Table 1:** Patient baseline characteristics.

	TKI monotherapy	Ipi+Nivo	Axi+Pembro
	n = 4183	n = 1555	n = 559
**Age, years, mean (SD)**	63.3 (11.4)	62.2 (10.9)	64.6 (10.8)*
**Sex, male (%)**	2974 (71)	1154 (72)	389 (70)
**Race, n (%)**			
**White**	3278(78)	1268 (80)	414 (74)*
**Black**	302 (7)	92 (6)	37 (7)
**Asian**	78 (2)	47 (3)	15 (3)
**Others/unknown**	525 (13)	180 (11)	93 (16)
**BMI, mean (SD)**	29.5 (6.32)	29.2 (6.2)	29.5 (6.71)
**Nephrectomy history, n(%)**	1293 (31)	592 (39)*	196 (35)
**Metastatic site, n(%)**			
**Lung**	1260 (30)	674 (43)*	163 (29)
**Liver**	395 (9)	197 (13)*	51 (9)
**Bone**	962 (23)	475 (30)*	114 (20)
**Brain**	125 (7)	169 (11)*	30 (5)
**Comorbidity, n(%)**			
**Diabetes Mellitus**	1009 (24)	488 (31)*	158 (28)
**Hypertension**	2249 (54)	980 (63)*	334 (60)*
**Cerebrovascular disease**	411 (10)	244 (16)*	71 (13)*
**Ischemia heart disease**	754 (18)	427 (27)*	112 (20)

Axi+Pembro: axitinib plus pembrolizumab; BMI: body mass index; Ipi+Nivo: ipilimumab plus nivolumab; SD: standard deviation; TKI: tyrosin kinase inhibitors.

* Statistical difference from TKI monotherapy cohort.

### Trends and sequences

**Fig 1:**
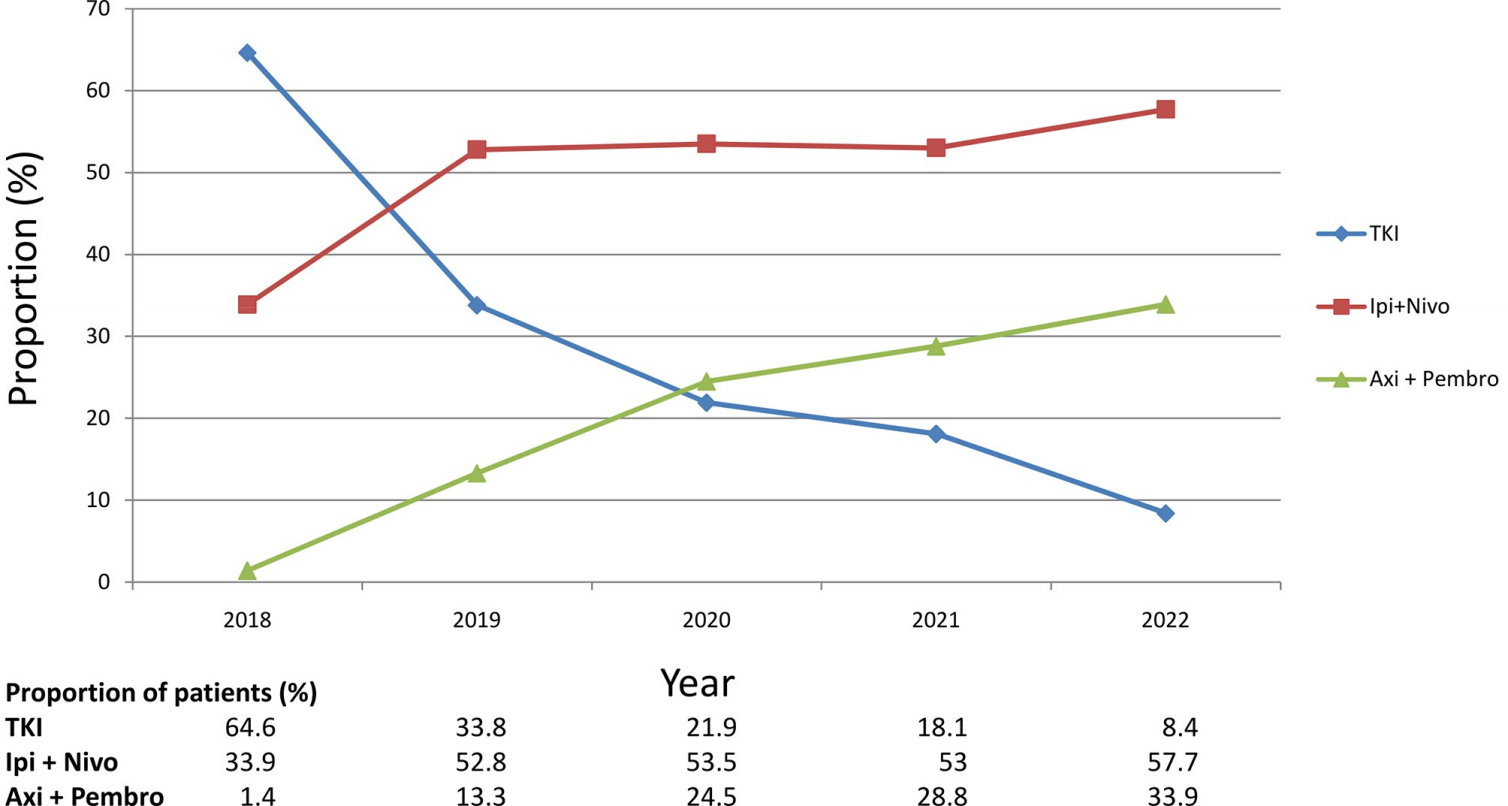
Trends of first-line treatment for metastatic renal cell carcinoma. Axi+Pembro: axitinib plus pembrolizumab; Ipi+Nivo: ipilimumab plus nivolumab; TKI: tyrosin kinase inhibitors.

The treatment trends regarding first-line systemic therapy for mRCC are shown in Fig 1. There has been an increased use of IO combination therapies (ipilimumab plus nivolumab and pembrolizumab plus axitinib) since 2018.

**Fig 2:**
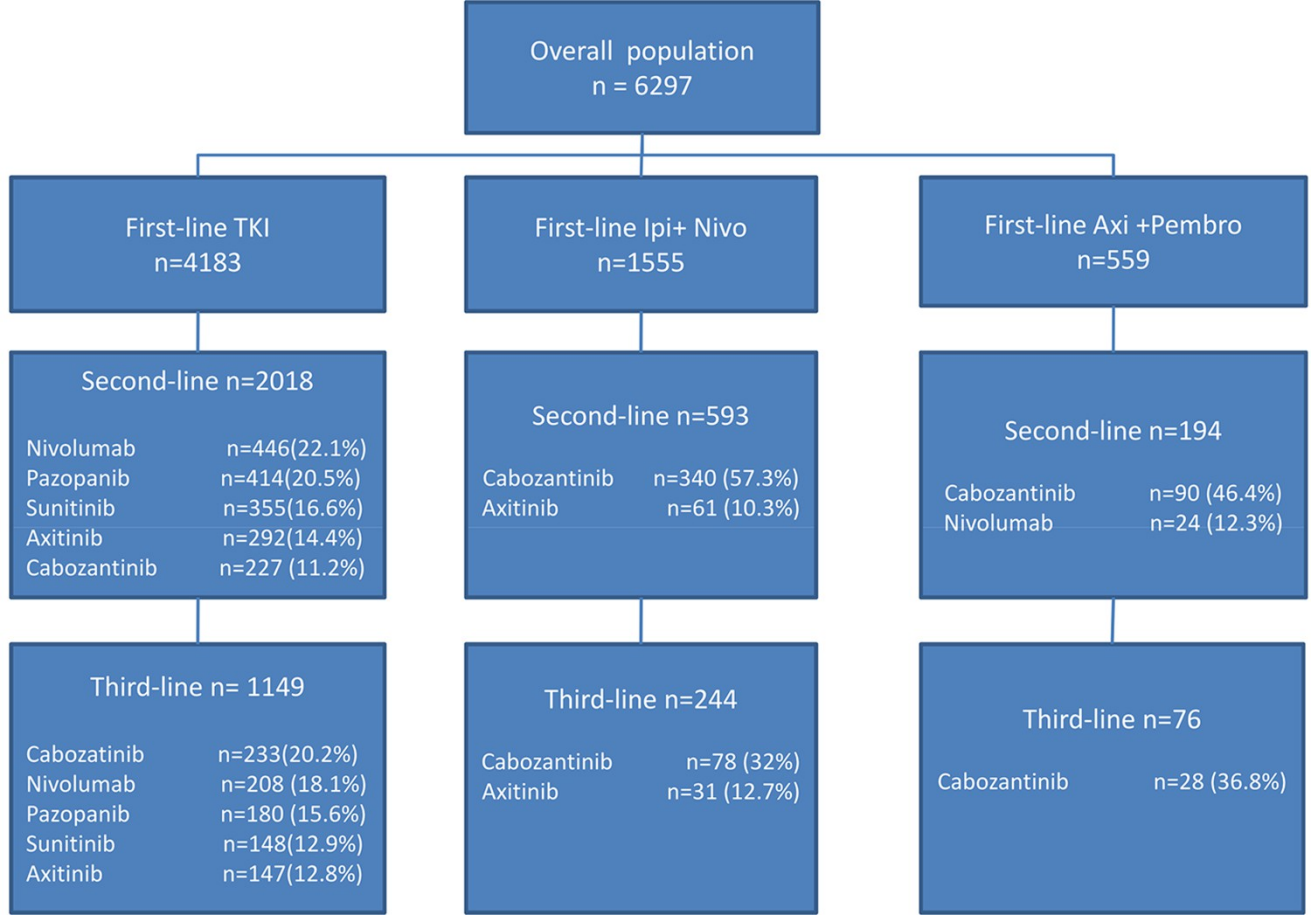
Sequences of systemic treatment for metastatic renal cell carcinoma. Axi+Pembro: axitinib plus pembrolizumab; Ipi+Nivo: ipilimumab plus nivolumab; TKI: tyrosin kinase inhibitors.

In all patients, 2805 (44.5%) received second-line systemic therapy. For TKI monotherapy cohort, frequencies of second-line treatments used were nivolumab(22.1%), pazopanib (20.5%), sunitinib (16.6%), and axitinib (14.4%). For ipilimumab plus nivolumab cohort, they were cabozatinib(57.3%) and axitinib (10.3%); and pembrolizumab plus axitinib cohort, cabozatinib (46.4%) and nivolumab (12.3%). In total, 1469 (23.3%) patients received third-line therapy for mRCC with cabozatinib(23.1%)being the most commonly used third-line treatment. Treatment sequences among different cohorts are shown in Fig 2.

### Outcomes

**Fig 3:**
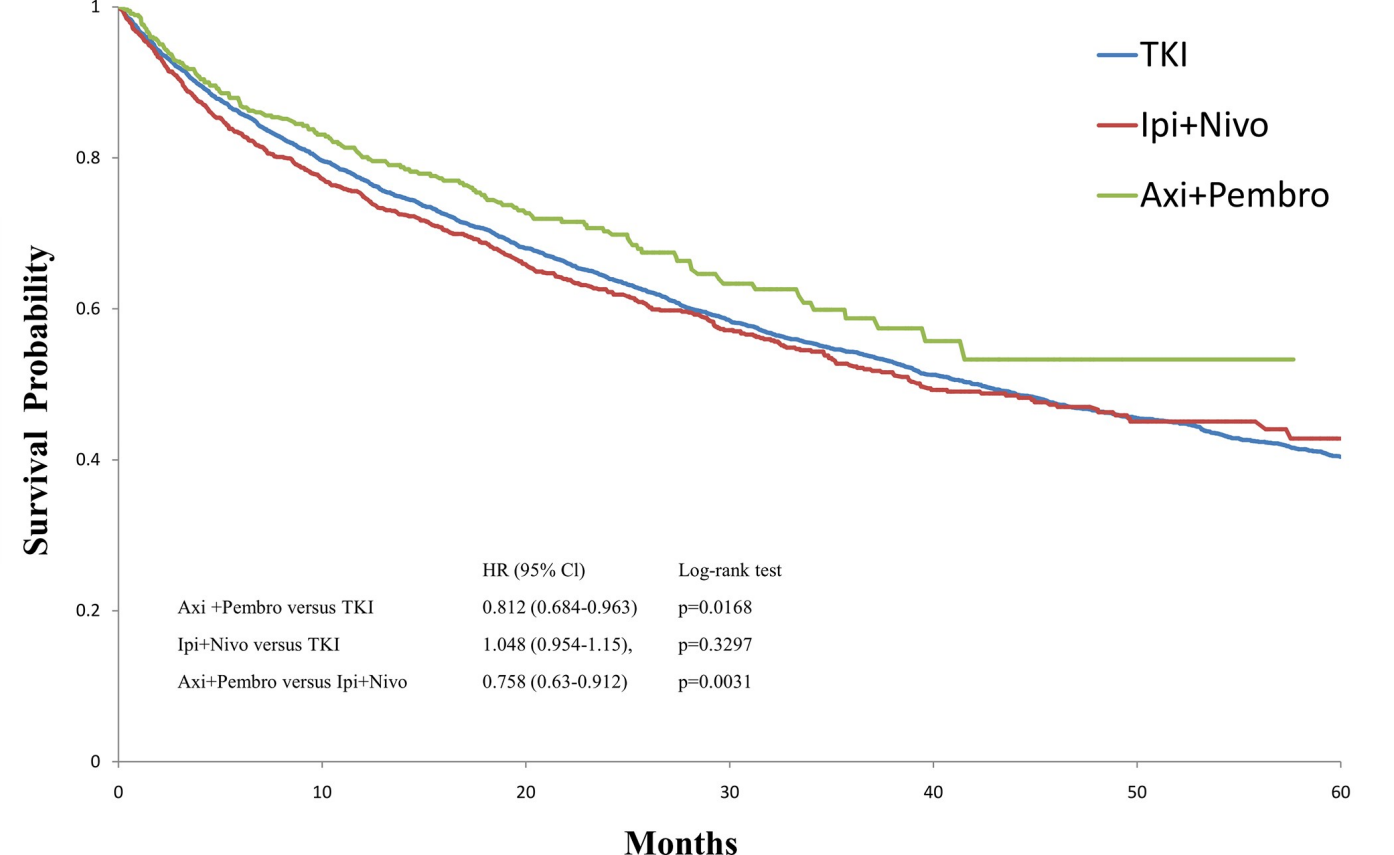
Kaplan-Meier survival curve for overall survival among tyrosin kinase inhibitors monotherapy (bleu), ipilimumab plus nivolumab (red), and axitinib plus pembrolizumab (green) cohort. Axi+Pembro: axitinib plus pembrolizumab; CI: confidence interval; HR: hazard ration; Ipi+Nivo: ipilimumab plus nivolumab; TKI: tyrosin kinase inhibitors.

Mean follow-up time (SD) was 35.3 (36.6) months for the TKI monotherapy cohort, 19.7 (17.2) months for the ipilimumab plus nivolumab cohort, and 16.7 (12.6) months for the pembrolizumab plus axitinib cohort. The survival probability at 12 months was 76.9% [95% confidence interval (CI) 75.6–78.2] for the TKI monotherapy cohort, 74.2% (95% CI 71.8–76.5) for the ipilimumab plus nivolumab cohort, and80.6%(95CI: 76.7–83.8) for the pembrolizumab plus axitinib cohort. Median OS was 42.2 months for the TKI monotherapy cohort, 39.7monthsfor the ipilimumab plus nivolumab cohort, and not reached for the pembrolizumab plus axitinib cohort. In comparison with the TKI monotherapy cohort, patients in the pembrolizumab plus axitinib cohort showed survival benefit (log-rank *p* = 0.0168) in OS, but not the case in the ipilimumab plus nivolumab cohort (Fig 3).

**Fig 4:**
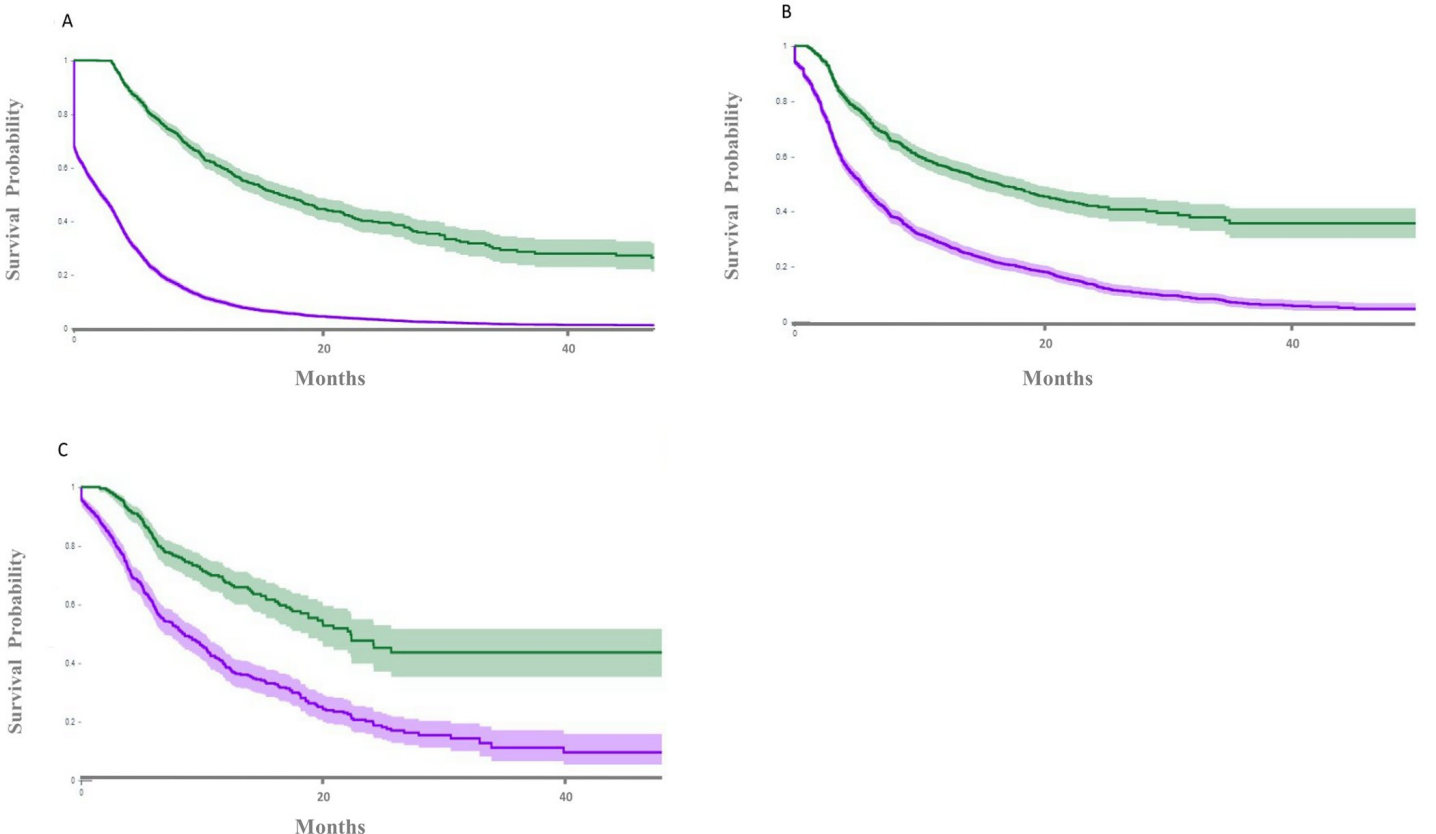
Kaplan-Meier survival curve for time on treatment (purple) and time to next treatment (green) for tyrosin kinase inhibitors monotherapy (A), ipilimumab plus nivolumab(B), and Axitinib plus pembrolimab cohort(C). Lighter area around the dark line means 95% confidence interval.

Median ToT was 2.5 months for the TKI monotherapy group, 5.4 months for the ipilimumab plus nivolumab group, and 8.3 months for the pembrolizumab plus axitinib group. Similarly, median TNT was16.6 months for both the TKI monotherapy and ipilimumab plus nivolumab cohorts, and 22.1 months for the pembrolizumab plus axitinib cohort (Fig 4). Clinical outcomes among different cohorts are summarized in Table 2.

**Table 2:** Clinical outcomes among different cohorts.

	Survival probability at 12 months, % (95% CI)	Median OS, months	Median time on treatment, months	Median time to next treatment, months
TKI monotherapy	76.9 (75.6–78.2)	42.2	2.5	16.6
Ipi+Nivo	74.2 (71.8–76.5)	39.7	5.4	16.6
Axi+Pembro	80.6 (76.7–83.8)	NR	8.3	22.1

Axi+Pembro: axitinib plus pembrolizumab; CI: confidence interval Ipi+Nivo: ipilimumab plus nivolumab; NR: not reached; OS: overall survival; SD: standard deviation; TKI: tyrosin kinase inhibitors.

## Discussion

The treatment landscape for mRCC changed a lot since the introduction of IO-based combination therapy. In the present study, we used TriNetx network database to conduct a cohort study with a large population ofmRCC patients who received first-line systemic therapy. We evaluated the treatment sequences and outcomes of IO-based therapies in a real-world setting.

Clinical trials are generally associated with highly selected patients in a well controlled circumstance to avoid biases, which may not reflect the real-world clinical practice. In this study, we conducted a retrospective cohort study to show their real-world treatment patterns and outcomes for patients with mRCC. For baseline characteristics, the ipilimumab plus nivolumab cohort was associated with more instances of distant metastases and co-mobidities in comparison with other groups. TKI monotherapy cohort and pembrolizumab plus axitinib cohort showed similar baseline characteristics. A possible explanation of such discrepancy might be that ipilimumab plus nivolumab was used for intermittent- and poor-risk tumors, and patients in this cohort had more risk factors and therefore poor prognosis.

The US Food and Drug Administration approved the use of ipilimumab plusnivolumab in 2018 and pembrolizumab plus axitinib in 2019 for the treatment of advanced RCC. We observed a trend toward an increasing use of ipilimumab plus nivolumab and pembrolizumab plus axitinib after the FDA approval. Literatures reported that second-line therapies are affected by the first-line treatments:patients received TKIs after IO-based therapies; patients received IO-based therapiesafterTKIs treatment [11, 13, 14]. The present study also observed a similar finding: for patients receiving ipilimumab plus nivolumabor pembrolizumab plus axitinib as first-line therapy, the most common second-line treatment was cabozantinib (54.6%). Among patients receiving TKIs as first-line therapy, the frequently used second-line treatments were nivolumab (22.1%), pazopanib (20.5%), sunitinib(16.6%), and axitinib (14.4%). The possible explanation for such discrepancy may be that our study included patients with mRCC between 2008 and 2022, partially reflecting the treatment patterns before the era of IO since nivolumab was first introduced as second-line therapy in 2015 [15].

According to previous studies, TOT and TNT are highly correlated with progression free survival [16, 17]. We found that the pembrolizumab plus axitinib cohort had the longest TOT and TNT. In additional, the pembrolizumab plus axitinib cohort was associated with a better OS compared with TKI monotherapy and ipilimumab plus nivolumab cohort. Our findings were consistent with the previous literatures regarding pembrolizumab plus axitinib being the most preferable first-line agents [11, 18, 19].

Contrary to the previous studies [20, 21], which reported pembrolizumab plus axitinib and ipilimumab plus nivolumab had similar outcomes, in this study, we found that the ipilimumab plus nivolumab cohort presented similar OS compared with the TKI montherapy cohort and their OS was worse than the pembrolizumab plus axitinib cohort. This discrepancy might be attributed to the fact that patients in the ipilimumab plus nivolumab cohort carried more instances of distant metastases and co-mobidities than the other two groups. Furthermore, ipilimumab plus nivolumab was not approved for regular use in the favorable risk group, indicating that patients with intermittent- and poor-risk tumors formed the majority of this cohort.

Our study has several limitations. First, its retrospective nature and non-randomization of our study are subject to selection bias. Second, several factors, such as IMDC risk stratification, clinical symptoms, reason of discontinuation and histopathological characteristics, were associated with prognosis and quality of life [12, 22–24]. The information and a Cox regression model analysis were not available from the database. Lastly, the TKI scohort in our study included patients over the past 10 years. Treatment patterns changed between different time periods, which may lead to bias in outcome comparisons. However, this study summarized the treatment sequences in the past decade for this cohort and showed treatment trends in the era of IO.

## Conclusion

We found a temporal trend toward using first-line IO-based therapy for patients with mRCC in a real-world clinical practice. Axitinib plus pembrolizumuab cohort showed survival benefits over TKI and ipilimumab plus nivolumab cohorts, while patients in the ipilimumab plus nivolumab cohort had more risk factors for poorer outcomes.

## Supporting information

S1 Table ICD-10-CM codes and corresponding diagnosis. (DOCX)

S1 File Data for Kaplan-Meier survival analysis. (XLSX)
