# Supplementary material for: Real world treatment sequences and outcomes for metastatic renal cell carcinoma
Source: PLoS One. 2023 Nov 22;18(11):e0294039. doi: 10.1371/journal.pone.0294039 (PMC10664936; doi:10.1371/journal.pone.0294039)
Supplement: S1 Table — (DOCX) [file pone.0294039.s001.docx]

**Supplemental Table 1. ICD-10-CM codes and corresponding diagnosis.**

|  | |
| --- | --- |
| ICD-10 code | Diagnosis |
| C64 | Malignant neoplasm of kidney, except renal pelvis · |
| C78.0 | Secondary malignant neoplasm of lung |
| C78.7 | Secondary malignant neoplasm of liver and intrahepatic bile duct |
| C79.31 | Secondary malignant neoplasm of brain |
| C79.5 | Secondary malignant neoplasm of bone and bone marrow |
| I10-I16 | Hypertensive diseases |
| I20-I25 | Ischemic heart diseases |
| E08-E13 | Diabetes Mellitus |
| I60-I69 | Cerebrovasculaelr disease |
| Z90.5 | Acquired absence of kidney |
| ICD-10-CM: International Classification of Diseases; Tenth Revision, Clinical Modification | |
