# Supplementary material for: Real world treatment sequences and outcomes for metastatic renal cell carcinoma
Source: PLoS One. 2023 Nov 22;18(11):e0294039. doi: 10.1371/journal.pone.0294039 (PMC10664936; doi:10.1371/journal.pone.0294039)
Supplement: Attachment — Submitted filename: Response to Reviewers.docx [file pone.0294039.s003.docx]

Reviewer #1: The study assesses a current, timely topic in renal cancer. An impressive number of patients and cases have been included. A linguistic revision is required.

We believe this article is suitable for publication in the journal although major revisions are needed. The main strengths of this paper are that it addresses an interesting and very timely question and provides a clear answer, with some limitations. Certainly, the authors should better highlight the limitations of the current paper.

- The background of the changing scenario of medical treatment in renal cancer should be better discussed, and some recent papers regarding this topic should be included ( PMID:36414800; PMID: 35031442; PMID: 36633661; PMID: 33880963).

We revised the manuscript and discussed the papers regarding renal cancers in the discussion and limitations sections. Please check it.

Reviewer #2: The article is interesting for the scientific community, but the conclusion was compromised by limitations.

The work needs to be revised and rewritten.

Attached parts of the text with comments:

Abstract

“Materials and Methods

Patients with metastatic renal cell carcinoma on the database of TriNetX network

were enrolled.”

It is necessary to inform the beginning and end period of data entry relating to the study.

The beginning and end period of data relating to the study were added in the revised manuscript. Please check it.

Patients were stratified according to the first-line treatment and their clinical outcomes were evaluated.

It is necessary to inform which assessments were carried out.

Assessments carried out for metastatic renal cell carcinoma were added in the revised manuscript. Please check it.

Results

Totally, 4183 received tyrosine kinase inhibitor monotherapy, 1555 received

ipilimumab plus nivolumab, and 559 received axitinib plus pembrolizumab.

It is necessary to inform start and end dates.

The dates were added in the revised manuscript. Please check it.

Median time on treatment was ...

It is necessary to inform how this calculation was made.

Overall survival, time on treatment and time to next treatment were evaluated using Kaplan-Meier method. These were added in the revised manuscript. Please check it.

Introduction

With the introduction of tyrosine kinase inhibitor (TKI) and immune-oncology (IO)

agent, the first-line systemic therapy for advanced renal cell carcinoma (RCC) has

evolved in recent years [1-9]. The current European Association of Urology

Guidelines ...

Reference 3 is from 2011 (more than 10 years), needs to be exchanged for a more recent reference.

The reference was changed. Please check it.

However, only a few studies reported real-world data regarding treatment pattern and clinical outcome of combination therapies on RCC.

Needs reference.

The references were added in the revised manuscript. Please check it.

Materials and Methods

Data source

We conducted a retrospective analysis of patients …

Retrospective analysis requires putting dates.

Dates were added in the revised manuscript. Please check it.

Limitations

Lastly, the TKIs cohort in our study included patients over the past 10 years. Treatment patterns changed between different time periods, which may lead to bias in outcome comparisons.

Conclusion

We found a temporal trend toward using first-line IO-based therapy for patients with

mRCC in a real-world clinical practice.

Treatment patterns changed between different time periods versus real-world clinical practice.

Limitations induce doubts regarding the completion of the work developed.

Though the TKIs cohort in our study included patients over the past 10 years, our study summarized the treatment sequences in the past decade for this cohort and showed treatment trends in the era of IO.

This was added in the revised manuscript. Please check it.
