# Supplementary material for: Real world treatment sequences and outcomes for metastatic renal cell carcinoma
Source: PLoS One. 2023 Nov 22;18(11):e0294039. doi: 10.1371/journal.pone.0294039 (PMC10664936; doi:10.1371/journal.pone.0294039)
Supplement: Peer review history

# Peer review: Decision Letter 0

**DOI:** 10.1371/journal.pone.0294039.r001

**Authors:** Alessandro Rizzo

13 Oct 2023

PONE-D-23-24098Real world treatment sequences and outcomes for metastatic renal cell carcinomaPLOS ONE

Dear Dr. Lai,

Thank you for submitting your manuscript to PLOS ONE. After careful consideration, we feel that it has merit but does not fully meet PLOS ONE’s publication criteria as it currently stands. Therefore, we invite you to submit a revised version of the manuscript that addresses the points raised during the review process.

Please submit your revised manuscript by Nov 27 2023 11:59PM. If you will need more time than this to complete your revisions, please reply to this message or contact the journal office at plosone@plos.org. When you're ready to submit your revision, log on to https://www.editorialmanager.com/pone/ and select the 'Submissions Needing Revision' folder to locate your manuscript file.

Please include the following items when submitting your revised manuscript:

- A rebuttal letter that responds to each point raised by the academic editor and reviewer(s). You should upload this letter as a separate file labeled 'Response to Reviewers'.
- A marked-up copy of your manuscript that highlights changes made to the original version. You should upload this as a separate file labeled 'Revised Manuscript with Track Changes'.
- An unmarked version of your revised paper without tracked changes. You should upload this as a separate file labeled 'Manuscript'.

If you would like to make changes to your financial disclosure, please include your updated statement in your cover letter. Guidelines for resubmitting your figure files are available below the reviewer comments at the end of this letter.

If applicable, we recommend that you deposit your laboratory protocols in protocols.io to enhance the reproducibility of your results. Protocols.io assigns your protocol its own identifier (DOI) so that it can be cited independently in the future. For instructions see: https://journals.plos.org/plosone/s/submission-guidelines#loc-laboratory-protocols. Additionally, PLOS ONE offers an option for publishing peer-reviewed Lab Protocol articles, which describe protocols hosted on protocols.io. Read more information on sharing protocols at https://plos.org/protocols?utm_medium=editorial-email&utm_source=authorletters&utm_campaign=protocols.

We look forward to receiving your revised manuscript.

Kind regards,

Alessandro Rizzo

Academic Editor

PLOS ONE

Journal requirements:

When submitting your revision, we need you to address these additional requirements.

1. Please ensure that your manuscript meets PLOS ONE's style requirements, including those for file naming. The PLOS ONE style templates can be found at

https://journals.plos.org/plosone/s/file?id=wjVg/PLOSOne_formatting_sample_main_body.pdf and

https://journals.plos.org/plosone/s/file?id=ba62/PLOSOne_formatting_sample_title_authors_affiliations.pdf

2. Thank you for stating the following financial disclosure:

“The funders had no role in study design, data collection and analysis, decision to publish, or preparation of the manuscript.”

At this time, please address the following queries:

a) Please clarify the sources of funding (financial or material support) for your study. List the grants or organizations that supported your study, including funding received from your institution.

b) State what role the funders took in the study. If the funders had no role in your study, please state: “The funders had no role in study design, data collection and analysis, decision to publish, or preparation of the manuscript.”

c) If any authors received a salary from any of your funders, please state which authors and which funders.

d) If you did not receive any funding for this study, please state: “The authors received no specific funding for this work.”

Please include your amended statements within your cover letter; we will change the online submission form on your behalf.

3. In your Data Availability statement, you have not specified where the minimal data set underlying the results described in your manuscript can be found. PLOS defines a study's minimal data set as the underlying data used to reach the conclusions drawn in the manuscript and any additional data required to replicate the reported study findings in their entirety. All PLOS journals require that the minimal data set be made fully available. For more information about our data policy, please see http://journals.plos.org/plosone/s/data-availability.

Upon re-submitting your revised manuscript, please upload your study’s minimal underlying data set as either Supporting Information files or to a stable, public repository and include the relevant URLs, DOIs, or accession numbers within your revised cover letter. For a list of acceptable repositories, please see http://journals.plos.org/plosone/s/data-availability#loc-recommended-repositories. Any potentially identifying patient information must be fully anonymized.

Important: If there are ethical or legal restrictions to sharing your data publicly, please explain these restrictions in detail. Please see our guidelines for more information on what we consider unacceptable restrictions to publicly sharing data: http://journals.plos.org/plosone/s/data-availability#loc-unacceptable-data-access-restrictions. Note that it is not acceptable for the authors to be the sole named individuals responsible for ensuring data access.

We will update your Data Availability statement to reflect the information you provide in your cover letter.

[Note: HTML markup is below. Please do not edit.]

Reviewers' comments:

Reviewer's Responses to Questions

**Comments to the Author**

1. Is the manuscript technically sound, and do the data support the conclusions?

Reviewer #1: Partly

Reviewer #2: Partly

**********

2. Has the statistical analysis been performed appropriately and rigorously? 

Reviewer #1: Yes

Reviewer #2: I Don't Know

**********

3. Have the authors made all data underlying the findings in their manuscript fully available?

Reviewer #1: Yes

Reviewer #2: No

**********

4. Is the manuscript presented in an intelligible fashion and written in standard English?

Reviewer #1: No

Reviewer #2: Yes

**********

5. Review Comments to the Author

Reviewer #1: The study assesses a current, timely topic in renal cancer.An impressive number of patients and cases have been included.

A linguistic revision is required.

We believe this article is suitable for publication in the journal although major revisions are needed. The main strengths of this paper are that it addresses an interesting and very timely question and provides a clear answer, with some limitations. Certainly, the authors should better highlight the limitations of the current paper.

- The background of the changing scenario of medical treatment in renal cancer should be better discussed, and some recent papers regarding this topic should be included ( PMID: 36414800; PMID: 35031442; PMID: 36633661; PMID: 33880963).

Reviewer #2: The article is interesting for the scientific community, but the conclusion was compromised by limitations.

The work needs to be revised and rewritten.

Attached parts of the text with comments:

Abstract

“Materials and Methods

Patients with metastatic renal cell carcinoma on the database of TriNetX network

were enrolled.”

It is necessary to inform the beginning and end period of data entry relating to the study.

Patients were stratified according to the first-line treatment and their

clinical outcomes were evaluated.

It is necessary to inform which assessments were carried out.

Results

Totally, 4183 received tyrosine kinase inhibitor monotherapy, 1555 received

ipilimumab plus nivolumab, and 559 received axitinib plus pembrolizumab.

It is necessary to inform start and end dates.

Median time on treatment was ...

It is necessary to inform how this calculation was made.

Introduction

With the introduction of tyrosine kinase inhibitor (TKI) and immune-oncology (IO)

agent, the first-line systemic therapy for advanced renal cell carcinoma (RCC) has

evolved in recent years [1-9]. The current European Association of Urology

Guidelines ...

Reference 3 is from 2011 (more than 10 years), needs to be exchanged for a more recent reference.

However, only a few studies reported real-world data

regarding treatment pattern and clinical outcome of combination therapies on RCC.

Needs reference.

Materials and Methods

Data source

We conducted a retrospective analysis of patients …

Retrospective analysis requires putting dates.

Limitations

Lastly, the TKIs cohort in our study included patients over the past 10 years. Treatment patterns changed between different time periods, which may lead to bias in outcome comparisons.

Conclusion

We found a temporal trend toward using first-line IO-based therapy for patients with

mRCC in a real-world clinical practice.

Treatment patterns changed between different time periods versus real-world clinical practice.

Limitations induce doubts regarding the completion of the work developed.

**********

6. PLOS authors have the option to publish the peer review history of their article (what does this mean?). If published, this will include your full peer review and any attached files.

If you choose “no”, your identity will remain anonymous but your review may still be made public.

**Do you want your identity to be public for this peer review?** For information about this choice, including consent withdrawal, please see our Privacy Policy.

Reviewer #1: No

Reviewer #2: No

**********

[NOTE: If reviewer comments were submitted as an attachment file, they will be attached to this email and accessible via the submission site. Please log into your account, locate the manuscript record, and check for the action link "View Attachments". If this link does not appear, there are no attachment files.]

While revising your submission, please upload your figure files to the Preflight Analysis and Conversion Engine (PACE) digital diagnostic tool, https://pacev2.apexcovantage.com/. PACE helps ensure that figures meet PLOS requirements. To use PACE, you must first register as a user. Registration is free. Then, login and navigate to the UPLOAD tab, where you will find detailed instructions on how to use the tool. If you encounter any issues or have any questions when using PACE, please email PLOS at figures@plos.org. Please note that Supporting Information files do not need this step.

# Author comment: Author response to Decision Letter 0

**DOI:** 10.1371/journal.pone.0294039.r002

22 Oct 2023

Reviewer #1: The study assesses a current, timely topic in renal cancer. An impressive number of patients and cases have been included. A linguistic revision is required.

We believe this article is suitable for publication in the journal although major revisions are needed. The main strengths of this paper are that it addresses an interesting and very timely question and provides a clear answer, with some limitations. Certainly, the authors should better highlight the limitations of the current paper.

- The background of the changing scenario of medical treatment in renal cancer should be better discussed, and some recent papers regarding this topic should be included ( PMID:36414800; PMID: 35031442; PMID: 36633661; PMID: 33880963).

We revised the manuscript and discussed the papers regarding renal cancers in the discussion and limitations sections. Please check it.

Reviewer #2: The article is interesting for the scientific community, but the conclusion was compromised by limitations.

The work needs to be revised and rewritten.

Attached parts of the text with comments:

Abstract

“Materials and Methods

Patients with metastatic renal cell carcinoma on the database of TriNetX network

were enrolled.”

It is necessary to inform the beginning and end period of data entry relating to the study.

The beginning and end period of data relating to the study were added in the revised manuscript. Please check it.

Patients were stratified according to the first-line treatment and their clinical outcomes were evaluated.

It is necessary to inform which assessments were carried out.

Assessments carried out for metastatic renal cell carcinoma were added in the revised manuscript. Please check it.

Results

Totally, 4183 received tyrosine kinase inhibitor monotherapy, 1555 received

ipilimumab plus nivolumab, and 559 received axitinib plus pembrolizumab.

It is necessary to inform start and end dates.

The dates were added in the revised manuscript. Please check it.

Median time on treatment was ...

It is necessary to inform how this calculation was made.

Overall survival, time on treatment and time to next treatment were evaluated using Kaplan-Meier method. These were added in the revised manuscript. Please check it.

Introduction

With the introduction of tyrosine kinase inhibitor (TKI) and immune-oncology (IO)

agent, the first-line systemic therapy for advanced renal cell carcinoma (RCC) has

evolved in recent years [1-9]. The current European Association of Urology

Guidelines ...

Reference 3 is from 2011 (more than 10 years), needs to be exchanged for a more recent reference.

The reference was changed. Please check it.

However, only a few studies reported real-world data regarding treatment pattern and clinical outcome of combination therapies on RCC.

Needs reference.

The references were added in the revised manuscript. Please check it.

Materials and Methods

Data source

We conducted a retrospective analysis of patients …

Retrospective analysis requires putting dates.

Dates were added in the revised manuscript. Please check it.

Limitations

Lastly, the TKIs cohort in our study included patients over the past 10 years. Treatment patterns changed between different time periods, which may lead to bias in outcome comparisons.

Conclusion

We found a temporal trend toward using first-line IO-based therapy for patients with

mRCC in a real-world clinical practice.

Treatment patterns changed between different time periods versus real-world clinical practice.

Limitations induce doubts regarding the completion of the work developed.

Though the TKIs cohort in our study included patients over the past 10 years, our study summarized the treatment sequences in the past decade for this cohort and showed treatment trends in the era of IO. 

This was added in the revised manuscript. Please check it.

Attachment Submitted filename: Response to Reviewers.docx Click here for additional data file.

# Editor report: Decision Letter 1

**DOI:** 10.1371/journal.pone.0294039.r003

**Authors:** Alessandro Rizzo

24 Oct 2023

Real world treatment sequences and outcomes for metastatic renal cell carcinoma

PONE-D-23-24098R1

Dear Dr. Lai,

We’re pleased to inform you that your manuscript has been judged scientifically suitable for publication and will be formally accepted for publication once it meets all outstanding technical requirements.

Within one week, you’ll receive an e-mail detailing the required amendments. When these have been addressed, you’ll receive a formal acceptance letter and your manuscript will be scheduled for publication.

An invoice for payment will follow shortly after the formal acceptance. To ensure an efficient process, please log into Editorial Manager at http://www.editorialmanager.com/pone/, click the 'Update My Information' link at the top of the page, and double check that your user information is up-to-date. If you have any billing related questions, please contact our Author Billing department directly at authorbilling@plos.org.

If your institution or institutions have a press office, please notify them about your upcoming paper to help maximize its impact. If they’ll be preparing press materials, please inform our press team as soon as possible -- no later than 48 hours after receiving the formal acceptance. Your manuscript will remain under strict press embargo until 2 pm Eastern Time on the date of publication. For more information, please contact onepress@plos.org.

Kind regards,

Alessandro Rizzo

Academic Editor

PLOS ONE

# Editor report: Acceptance letter

**DOI:** 10.1371/journal.pone.0294039.r004

**Authors:** Alessandro Rizzo

13 Nov 2023

PONE-D-23-24098R1 

Real world treatment sequences and outcomes for metastatic renal cell carcinoma 

Dear Dr. Lai:

I'm pleased to inform you that your manuscript has been deemed suitable for publication in PLOS ONE. Congratulations! Your manuscript is now with our production department. 

If your institution or institutions have a press office, please let them know about your upcoming paper now to help maximize its impact. If they'll be preparing press materials, please inform our press team within the next 48 hours. Your manuscript will remain under strict press embargo until 2 pm Eastern Time on the date of publication. For more information please contact onepress@plos.org.

If we can help with anything else, please email us at plosone@plos.org. 

Thank you for submitting your work to PLOS ONE and supporting open access. 

Kind regards, 

PLOS ONE Editorial Office Staff

on behalf of

Dr. Alessandro Rizzo 

Academic Editor

PLOS ONE
